# Validation of a High Sampling Rate Inertial Measurement Unit for Acceleration During Running

**DOI:** 10.3390/s17091958

**Published:** 2017-08-25

**Authors:** Thomas Provot, Xavier Chiementin, Emeric Oudin, Fabrice Bolaers, Sébastien Murer

**Affiliations:** 1 EPF, 3 bis rue Lakanal, 92330 Sceaux, France; thomas.provot@epf.fr; 2 GRESPI, Université de Reims Champagne-Ardenne, 51687 Reims CEDEX 2, France; fabrice.bolaers@univ-reims.fr (F.B.); sebastien.murer@univ-reims.fr (S.M.); 3 neXXtep Technologies, 11 rue du Four, 51000 Châlons-en-Champagne, France; emeric.oudin@nexxtep-technologies.com

**Keywords:** inertial measurement unit, accelerometer, reliability, running

## Abstract

The musculo-skeletal response of athletes to various activities during training exercises has become a critical issue in order to optimize their performance and minimize injuries. However, dynamic and kinematic measures of an athlete’s activity are generally limited by constraints in data collection and technology. Thus, the choice of reliable and accurate sensors is crucial for gathering data in indoor and outdoor conditions. The aim of this study is to validate the use of the accelerometer of a high sampling rate (1344Hz) Inertial Measurement Unit (IMU) in the frame of running activities. To this end, two validation protocols are imposed: a classical one on a shaker, followed by another one during running, the IMU being attached to a test subject. For each protocol, the response of the IMU Accelerometer (IMUA) is compared to a calibrated industrial accelerometer, considered as the gold standard for dynamic and kinematic data collection. The repeatability, impact of signal frequency and amplitude (on shaker) as well as the influence of speed (while running) are investigated. Results reveal that the IMUA exhibits good repeatability. Coefficient of Variation CV is $1\%\left(8.58\pm 0.06\;m/s^{2}\right)$ on the shaker and $3\%\left(26.65\pm 0.69\;m/s^{2}\right)$ while running. However, the shaker test shows that the IMUA is affected by the signal frequency (error exceeds 10% beyond 80Hz), an observation confirmed by the running test. Nevertheless, the IMUA provides a reliable measure in the range 0–100 Hz, i.e., the most relevant part in the energy spectrum over the range 0–150 Hz during running. In our view, these findings emphasize the validity of IMUs for the measurement of acceleration during running.

## 1. Introduction

Studies on athletes’ health intend to improve human security and performance against stresses associated to sport activities [1,2,3]. Running related injuries, like the patellofemoral syndrome, iliotibial band friction syndrome, tibial stress syndrome, plantar fasciitis, Achilles tendonitis and meniscal injuries are very common [4,5,6]. Their causes are often manifold due to internal and external risk factors [7], yet a number of works have found that repeated impacts severely increase the risk of an overuse injury [8,9,10,11]. To gain more insight in the development of these injuries, many studies have used accelerometers to investigate the role of accelerations as well as impacts in the etiology of these injuries. Mizrahi et al. [12] measured acceleration at the tibial tuberosity to study fatigue of the human body. Similarly, Friesenbichler et al. [13] assessed muscle fatigue by placing the sensor on the triceps surae. In 2010, Lee et al. [14] evaluated the asymmetry of runners using an accelerometer at the sacrum (S1) level. Based on several studies, frequency and amplitude ranges of tibial acceleration can be determined. Fu et al. [15] concluded that ground surface has no effect on tibial acceleration (10.3 to 12.4 g across different surfaces). Hamill et al. [16] conclude that this acceleration decreases when the step frequency is 20% higher than the natural one. Gruber et al. [17] give characteristics for Peak Positive Tibial Acceleration (PPA) (which amplitude lies between 3.87 and 5.07 g) and the peak power for low (range 6.4–7.2 Hz) as well as high frequencies (range 10.7–14.3 Hz). These results are confirmed by the studies of Bobbert [18], which define two excited ranges: 4–8 Hz and 10–20 Hz.

Most of these studies have been performed in controlled environments, ensuring a more rigorous management of test parameters. However, it is clearly recognized that simulated conditions tend to alter the running pattern [14], giving rise to an increased number of outdoor test campaigns implementing portable measuring devices. Among these devices, we can cite the Inertial Measurement Unit (IMU), mostly used for navigation and consisting in an autonomous electronic board equipped with various sensors (e.g., accelerometer). Such a fully integrated device provides a relevant answer to the constraints of space, lightness and autonomy imposed by the measurement of human activities [14,19,20]. At first, IMUs were mostly used in lab conditions. For instance, in 2007, Elvin et al. [21] compare tibial acceleration obtained with wireless sensors and the ground reaction force during jump. Busa et al. [22] use accelerometers embedded in an electromyography system , to evaluate the tibial peak acceleration and estimate the visual feedback. But IMUs have also been used in outdoor conditions in order to collect field running parameters. In 2011, Chelius et al. [23] use IMUs during a six-day desert race competition on an athlete. Barrett et al. [24] define running events by means of an embedded IMU attached to the tibia. In 2010, Neville et al. [25] extend concepts about running speed and energy expenditure obtained in lab conditions to overground running. Recently, Horvais and Giandolini [26] observe the impact of different running patterns on the acceleration peak at the tibia. In 2015, Giandolini et al. [27] study the foot strike pattern on a world-class athlete during a 45km trail using the accelerometer of an IMU. These studies use in particular the IMU accelerometer, however this component is coupled with substructures. This influences largely its transfer function [28,29]. It is a systematic problem about how the component is embedded in an IMU. Thus, question remains as to the accuracy of these devices: does it compare to standard lab accelerometers?

The purpose of this study is to estimate the accuracy of a high sampling rate IMU for the measurement of acceleration during running. First, we will proceed with the validation of the IMU accelerometer (IMUA) on a shaker. Next, the validation will be carried out in real conditions during running. For both studies, the IMUA will be compared to an industrial calibrated piezoelectric accelerometer defined as gold standard.

## 2. Methods

### 2.1. Subject

One subject was recruited for this study (healthy 33-year-old man, at least 60km a week, equipped with personal running shoes). All procedures were reviewed and approved by the local Research Ethics Committee prior to the initiation of the research.

### 2.2. Protocols

This study is divided into two protocols: the validation of an IMU on shaker and during running. The IMU of interest is a Hikob^®^ Fox unit (General information: Hikob, Villeurbanne, France-dimensions: 45×36×17mm, mass: 22g. Accelerometer characteristics: sampling frequency: 1–1344 Hz, maximum magnitude: ±24 g).

**Figure 1:**
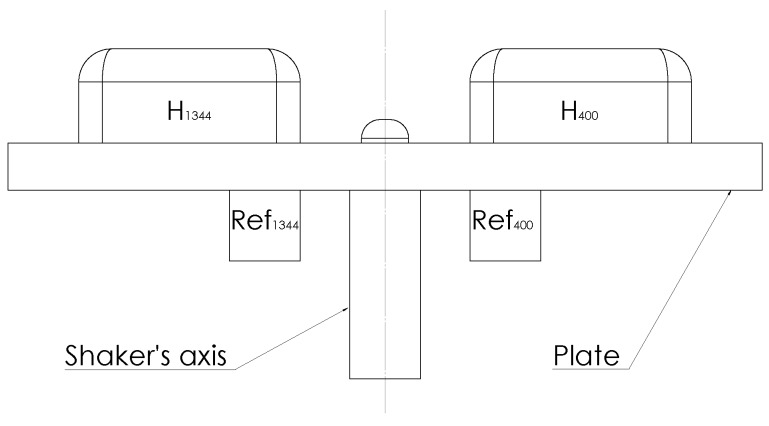
Test bench with the two IMUs (H${}_{1344}$, H${}_{400}$) and the two associated references (R${}_{1344}$, R${}_{400}$). Sensors are excited by a back and forth motion along the shaker axis.

The first protocol examines the validity of the IMUA tested on a shaker (Bruel & Kjaer Type 4809, frequency range: 10–20 kHz Naerum, Denmark) equipped with a plate where the sensors were fixed. In order to assess the impact of the sampling frequency on vibratory signals for the sport domain, two IMUs with two different accelerometer sampling frequencies were used in this study: H${}_{1344}$ and H${}_{400}$ for sampling rates of 1344Hz and 400Hz respectively. The maximum amplitude of the IMUA is adjustable as well and was set to ±4 g in order to maximise resolution. Both IMUs were attached face to face on the plate using cable ties. Each IMUA was associated and compared to a triaxial industrial accelerometer defined as gold standard (R${}_{1344}$, R${}_{400}$: Bruel & Kjaer 4525B, frequency range: 1–10,000 Hz. Naerum, Danemark). The two references were sampled at 2560Hz in order to measure a theoretical range of 0–1000 Hz [30]. Both sensors were glued using cyanoacrylate glue under the plate, beneath each associated IMU (see Figure 1). An OROS acquisition system (OROS, OR36, Grenoble, France) was used to record the acceleration measurements for the reference sensors, monitor the shaker and simulate a sine with given frequency and amplitude. This protocol falls into three steps in order to assess the following characteristics: accuracy and precision of the IMUA measures, impact of the vibration frequency on the IMUA response and impact of the vibration amplitude on the IMUA response. The first step investigated the precision and accuracy of the IMUA. Ten measurements were performed at 30Hz and at a Root Mean Square (RMS) amplitude of $7\;m/s^{2}$. A minimum interval of one hour was respected between each measurement. Temperature and humidity of the room were also recorded during each acquisition. The second step addressed the impact of the frequency of the measured vibration on the IMUA response. The IMUAs were tested on a broad frequency band ranging from 11 to 200Hz. Intrinsic limitations of the shaker led us to split the protocol into two parts. First, twenty-two frequencies were tested (20–25–30–35–40–45–50–60–70–80–90–100–110–120–130–140–150–160–170–180–190–200 Hz) at a RMS amplitude of $7\;m/s^{2}$. Then, five frequencies (11–13–15–17–19 Hz) were tested at a RMS amplitude of $2\;m/s^{2}$. The third step was dedicated to studying the impact of the vibration amplitude on the IMUA response. Seventeen amplitudes (1.5–2–2.5–3–3.5–4–4.5–5–6.5–7–7.5–8–8.5–9–9.5 m/s${}^{2}$) were tested with a constant frequency of 25Hz. This frequency was chosen since the tolerance sensitivity of the reference sensors at that frequency approaches 0%.

**Figure 2:**
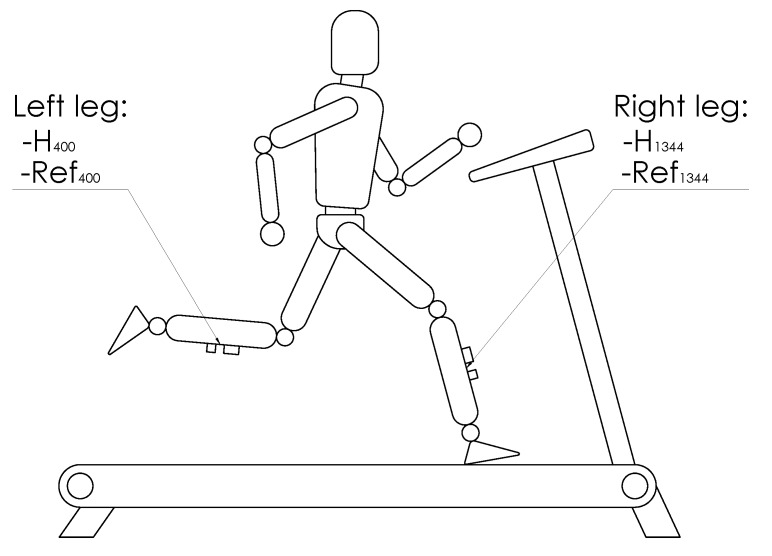
Sensors location on the subject’s legs, with both IMUs (H${}_{1344}$, H${}_{400}$) and the associated references (R${}_{1344}$, R${}_{400}$).

**Figure 3:**
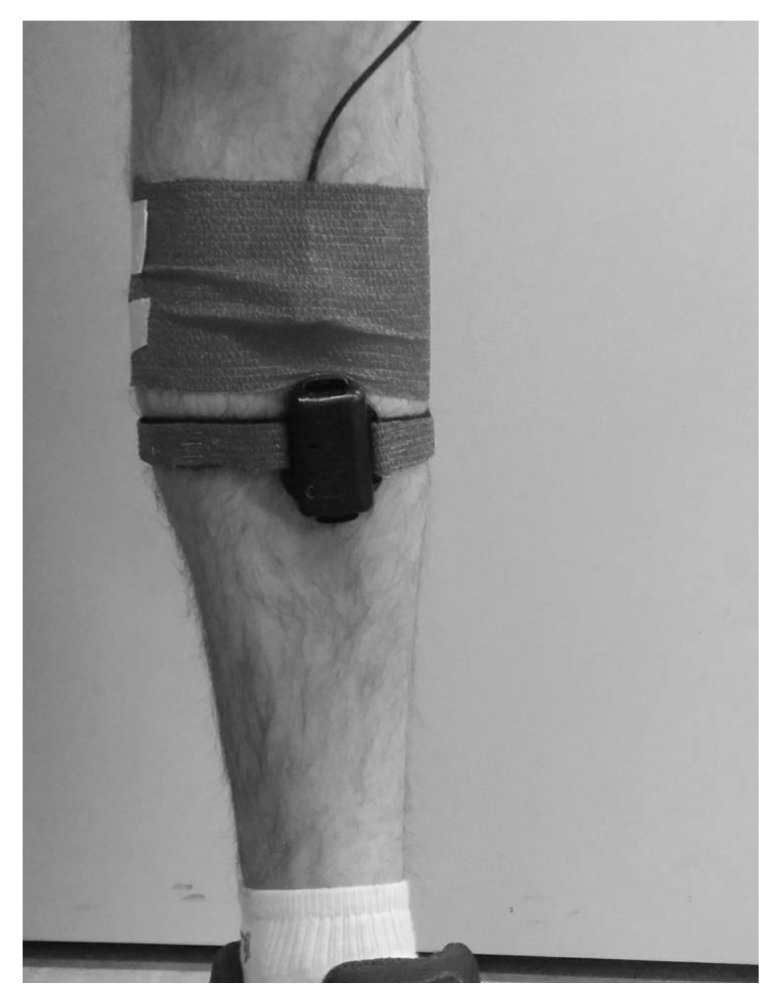
Attachment method of the IMU and the reference sensor on the subject’s leg.

**Figure 4:**
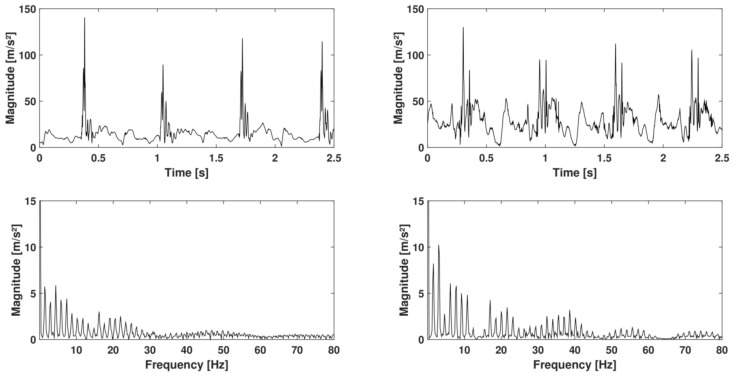
Temporal (**Top**) and frequency (**Bottom**) signals during running, measured by reference R${}_{1344}$ at 8km/h (total acceleration).

The second protocol was imposed during running. This protocol contained two steps dedicated to evaluating the repeatability of the IMUA measures in running conditions as well as the impact of the running speed on the IMUA response. Both steps were performed on a treadmill (LifeFitness, Rosemont, IL, USA). The subject was equipped on each tibia with an IMU and the reference accelerometer, similar to those used in the first protocol. In accordance with results from the literature, the sensors were located at the previously shaved, distal anteromedial aspect of the tibia, as close to the center of gravity of the leg as possible, following anthropometric data described by Winter [31]. This choice allowed meeting criteria such as a measurement on the human body with a minimum influence of soft tissues, a maximum range of amplitude and frequency, a measurement point close to the point of impact, and an area broad enough to accommodate an IMU and the reference accelerometer. To assess the impact of the sampling frequency on the running measurements, both legs are equipped. The right leg was equipped with an IMUA sampled at 1344Hz (H${}_{1344}$) with an amplitude of ±16 g and a reference sensor sampled at 2560Hz. The left leg was equipped with an IMUA sampled at 400Hz (H${}_{400}$) with an amplitude of ±16 g and a reference similar to the right leg (Figure 2). The reference sensors were attached to the tibia by means of an elastic band and a double-sided tape to prevent sensor rotation. The IMUs were fixed to the tibia using Velcro strip, held in the eyelets of the housings (Figure 3). The signals were collected by the OROS acquisition system for the references. For this study, the three axes of the IMUA and reference sensors are used in order to measure the complete running frequency range. The subject was first asked to warm up, practising two minutes of walking followed by five minutes of running at 12km/h (3.33m/s). During the measurements, the subject was asked to keep looking at a fixed point on the facing wall. The measurements were recorded over a period of five seconds, in the steady state. Ten measurements at 12km/h (endurance speed) were gathered during the first step, each one of them split into one minute of running at the selected speed followed by a two-minute rest. For the second step, six measurements at 8–10–12–14–16–18 km/h were performed. The five measures were composed by one minute of running at the selected speed followed by a two-minute rest. No removal or replacement of the accelerometers was performed to preclude the influence of the location [32]. Figure 4 depicts an example of measurement.

### 2.3. Data Analysis

Each measurement during the first protocol (on a shaker) had a duration of 5s and was represented by its RMS value in $m/s^{2}$ for the vertical axis. For the first step, the mean and standard deviation of the RMS values for the ten measurements were calculated for each sensor in order to estimate precision and accuracy of the sensors. The Coefficient of Variation (CV) of a sensor was calculated as the ratio of the standard deviation to the mean value. Regarding the other two steps, for each of the 27 frequencies and each of the 17 magnitudes, the RMS values from the IMUA were compared to those of their associated references. In order to study the impact of frequency and amplitude, results were presented as a relative difference between the IMUA and the associated reference.

For the second protocol (running condition), in an attempt to reduce the impact of the subject’s movement, each measurement was represented by a 5s signal in a steady state of the total acceleration, A(t) (see Equation (1)). Functions $A_{x}\left(t\right)$, $A_{y}\left(t\right)$ and $A_{z}\left(t\right)$ stand for the accelerations on each axis.

(1) $$A\left(t\right)=\sqrt{A_{x}{\left(t\right)}^{2}+A_{y}{\left(t\right)}^{2}+A_{z}{\left(t\right)}^{2}}$$

In this protocol, the first step consists in studying the repeatability of the measures. To this end, the RMS value of the signal was calculated for each of the ten tests and each sensor: its mean value, standard deviation and CV were computed. For the second step, as running does not present a particular frequency with a given magnitude but a large range of frequencies with variable magnitudes, the comparison between the IMUA and the reference was conducted by the study of similarity in the frequency domain. First, in order to compare the frequency domain of the different sensors, temporal signals were resampled to obtain a similar sampling rate. Then, spectral coherence was used on the resampled temporal signal. Spectral coherence (C) is a frequency function quantifying how signal (x) matches signal (y) at each frequency (f). It always satisfies 0≤C(f)≤1 and equals 1 in the ideal case. Spectral coherence is defined by Equation (2), where $P_{xx}\left(f\right)$ and $P_{yy}\left(f\right)$ respectively stand for the power spectral densities for signals (x) and (y); and $P_{xy}\left(f\right)$ for the cross power spectral density.

(2) $$C\left(f\right)=\frac{|P_{xy}\left(f\right){|}^{2}}{P_{xx}\left(f\right).P_{yy}\left(f\right)}$$

## 3. Results

**Figure 5:**
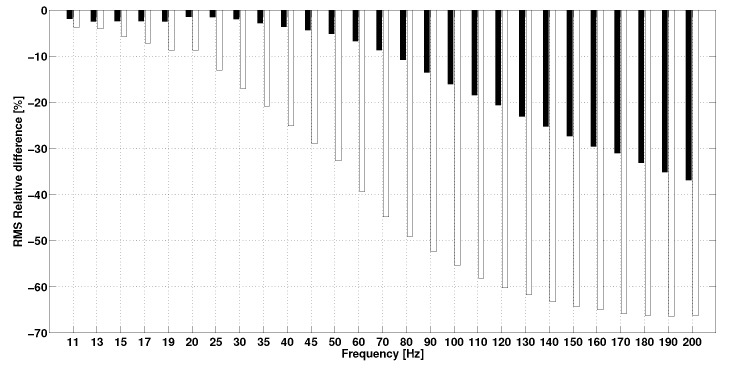
RMS relative difference between the IMUA and its reference vs. frequency (**Black**: H${}_{1344}$–R${}_{1344}$, **White**: H${}_{400}$–R${}_{400}$).

**Figure 6:**
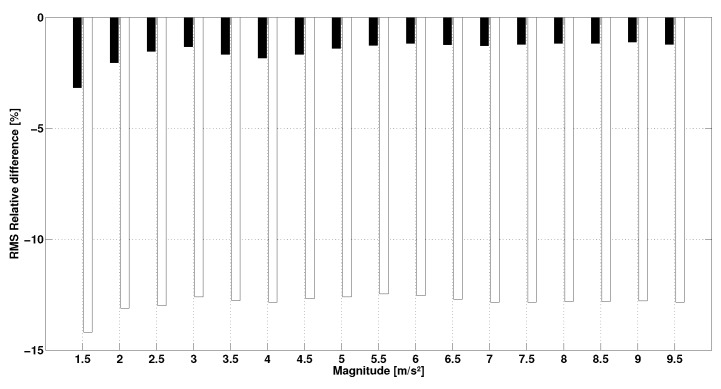
RMS relative difference between the IMUA and its reference vs. magnitude (**Black**: H${}_{1344}$–R${}_{1344}$, **White**: H${}_{400}$–R${}_{400}$).

In the first protocol, the measurements for the first step were spread over 24 h: room temperature fluctuated by 9% while hygrometry fluctuated by 17%. The results of this step show that the acceleration in terms of RMS value of H${}_{1344}$ ($8.58\pm 0.06\;m/s^{2}$) and H${}_{400}$ ($6.60\pm 0.07\;m/s^{2}$) has a CV of 1%. The results of the second test show the impact of the frequency on the sensor response (Figure 5). The figure displays the evolution of the relative difference in RMS between the IMUAs and their references as a function of frequency. It is observed that sensor H${}_{1344}$ underestimates acceleration. Relative error does not exceed 5% below 25Hz but keeps increasing up to 37% beyond 80Hz. Similarly, the H${}_{400}$ sensor also underestimates the value of acceleration. However, the error increases from 11Hz and exceeds 10% for frequencies over 25Hz. Beyond 140Hz, relative error stabilizes around 65%. The results of the last step on the impact of the magnitude on the sensor response are presented in Figure 6, where evolution of the relative difference in RMS between the IMUAs and references are plotted against the amplitude. Sensor H${}_{1344}$ seems to underestimate the amplitude of the measured signal. However, error remains stable around 2% on the range 1.5–9.5 m/pers${}^{2}$. The H${}_{400}$ sensor displays a similar behavior, but error is stable around 13% on the tested range.

**Figure 7:**
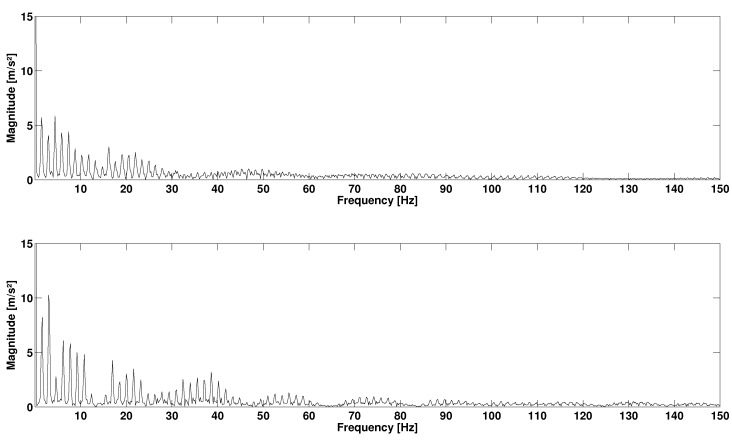
Total acceleration spectrum of the right leg reference sensor R${}_{1344}$ at 8km/h (**Top**) and 18km/h (**Bottom**).

**Figure 8:**
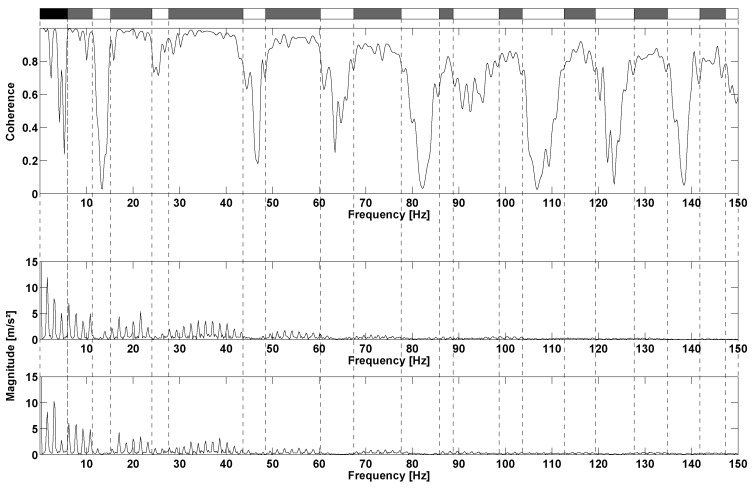
Spectral coherence between R${}_{1344}$ and H${}_{1344}$ at 18km/h: coherence (**top**), H${}_{1344}$ spectrum (**middle**) and R${}_{1344}$ spectrum (**bottom**). The black area is the frequency range presenting the stride frequency and first harmonics, white areas are lesser energy ranges and grey areas symbolize the IMUA response to excitation frequencies.

In the second protocol, the results of the repeatability step are also expressed in terms of RMS value: $22.65\pm 0.98\;m/s^{2}$ (CV = 2%) for H${}_{1344}$, $26.63\pm 0.61\;m/s^{2}$ (CV = 4%) for H${}_{400}$, $22.55\pm 0.84\;m/s^{2}$ (CV = 3%) for R${}_{1344}$, $26.66\pm 0.69\;m/s^{2}$ (CV = 4%) for R${}_{400}$. The total acceleration spectra of R${}_{1344}$ for two different speeds are presented in Figure 7. This figure shows that the running speed directly impacts the frequency signals. Higher peaks of amplitude are observed at high-speed running. Moreover, higher speeds tend to excite higher frequencies. At 8km/h, the relevant frequencies lie within the range 0–120 Hz, but exceed 150Hz at 18km/h. However, range 0–100 Hz generally represents the most important part of the spectrum with a larger amount of energy for cumulative frequencies. The coherence curves exhibit three types of frequency ranges (Figure 8). The first frequency range represents the stride frequency (fundamental) and two or three harmonics (black area). The second type of frequency ranges are those encompassing lesser energy (white area). These frequency ranges are characterized by lightly excited ranges (with low magnitude) and low spectral coherence. The third type of frequency ranges are those with a strong coherence (grey area). Remarkably, the most relevant ranges of frequencies display the strongest coherence and clearly represent the IMUA response to the signal frequencies.

## 4. Discussions

The validation protocols of the IMUA detailed above yield rather satisfactory results. On the shaker, IMUA displays a high level of precision (CV = 1%) despite the variation in room temperature and hygrometry. By contrast, accuracy turns out to be poor (+20% at a sampling frequency of 1344Hz, −5% at a sampling frequency of 400Hz). This unexpectedly low level is most likely due to the exciting frequency of the shaker. Indeed, for the frequency test (Figure 5), the 30Hz exciting frequency results in a discrepancy between each IMUA and its reference sensor. Yet taking account of industrial criteria for sensors validation (power gain ±1dB to ±3dB), such a level remains acceptable. The relative gap with the gold standard remains constant regardless of the acceleration magnitude, but increases with frequency to an extent that depends on the sampling rate of the IMUA. The effect of the signal frequency on the IMUA response as well as the impact of the sampling frequency on the bandwidth of the IMUA are also observed. Indeed, the bandwidth limit is found to lie between 80Hz and 170Hz for H${}_{1344}$; 25Hz and 50Hz for H${}_{400}$, depending on the desired industrial standard (power gain ±1dB to ±3dB). However, it is worth noting that the response of the IMUA to the signal frequency is also affected by the sensitivity of the reference sensors. According to the frequency response function provided by the manufacturer, the reference sensor has a tolerance of 0 to +4% on the range 5–200 Hz: this sensor is likely to slightly overstate the amplitude of the measured signal, while IMUAs highlight a tendency to underestimate the references. There is no denying that this sensitivity may be a limitation to the measurement in the field of other sport activities, where frequency range 0–200 Hz may appear.

**Figure 9:**
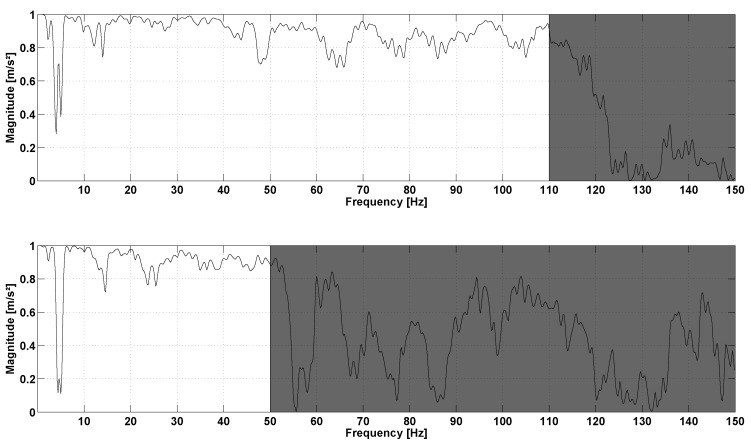
Spectral coherence between R${}_{1344}$ and H${}_{1344}$ at 12km/h (**Top**) and between R${}_{400}$ and H${}_{400}$ at 18km/h (**Bottom**).

During running activities, IMUAs prove to be both precise (CV = 3%) and accurate compared to the gold standards (0.4%), as confirmed by the first protocol. As regards the coherence curve, three areas can be distinguished. The black area is obtained for low frequencies, and two observations can be made. First, for some frequencies, there is a discrepancy in energy between the two sensors. Second, coherence remains low in this range, which may be accounted for by a difference in sensors sensitivity for low frequency signals. Moreover, the difference in mass and attachment method of the sensors on the skin between the IMUs and reference sensors may result in a movement of the soft tissues which could impact lower frequencies. Another possible reason for this is gravity, which is measured only by the IMUA. In a static reference system, gravity appears as a constant and alters only the 0Hz frequency. However, in the IMUA reference system – which follows leg movement – gravity will be detected cyclically on the different axes of the IMUA, at a frequency correlated with the kinematics of the leg [33]. Thus it is not particularly surprising that gravity will have an impact on a broad range of low frequencies that will be different for the IMUA and the reference. In view of these considerations, it is difficult to conclude whether the IMUA suffers from a sensitivity limitation or conveniently allows measuring gravity. The white areas represent low coherence, with various possible reasons as well. First, due to its resolution, the IMUA only provides an approximate value of the real signal at low amplitudes. In the same vein, the magnitude of the signal is not so different from the measurement noise: data transmitted by the IMUA are quite different from those of the reference, and do not present strong coherence. Finally, the grey areas are associated with good coherence. Observation of the corresponding ranges leads to state that the quality of the IMUA response decreases as frequency increases, which supports the results of the preliminary study on shaker. Discarding the range of the stride frequency and ranges with low energy, IMUA clearly highlight a different response depending on the sampling frequency (Figure 9). Results show that sensor H${}_{400}$ presents a strong spectral coherence for the range 0–50 Hz, but also that the response quality drops beyond that frequency. This range matches with the range determined in the literature [15,16,17,18], and therefore, the sampling rate should not be less than 400Hz during tests involving measurement of tibial acceleration. Moreover, as seen in the literature [25,26,27], this device could be used in real conditions. Thus, the feedback of IMUA could be used as a powerful tool to study and reduce impact loading during the activity [34] and the risk of injury [35].

According to Figure 7, the sampling frequency 400Hz seems unable to encompass a complete running spectrum, but only the 0–50 Hz range. Yet significant information could be located beyond 50Hz. Sensor H${}_{1344}$ presents a strong spectral coherence, at least for the range 0–100 Hz with contains the most excited range for the running spectrum. It is in contradiction with the studies implementing a second order Butterworth low-pass filter with a cut-off frequency of 60Hz (e.g., [36]), although peaks are likely to occur beyond this frequency. Even if this sampling frequency ensures proper representation of a complete running spectrum with no significant loss of information, the definition of the bandwidth of the IMUA could be influenced by various factors, including the mass of the device, the Velcro strip tension [37] coupled with the filter effect of human skin [38]. As a matter of fact, the viscoelastic behaviour of human soft tissue has been put forward in several studies to justify the absorption of high frequencies excited by a shock [30,39,40]. Moreover, it is worth noting that the IMUs were tested on shaker and during running with two different types of attachment means. It is recommended that future works should always test both IMUs with a Velcro strip. Finally, the discrepancy between the two sensors can be explained by their different locations on the tibia. For instance, Lucas-Cuevas et al. [41] actually emphasized on the difference in the temporal and spectral parameters depending on the position of the sensors, on the forehead, the proximal section and the section of the tibia.

## 5. Conclusions

This work validates the accelerometer of an IMU for the assessment of acceleration and vibration from dynamic data during running. The results of the studies on a shaker and during running show that the IMUA displays precise measurements with acceptable accuracy and supplies measures within a bandwidth of 0–100 Hz Beyond this range, although measures are still possible, the quality of the response is insufficient to allow correct interpretation of the results. Despite its narrower ranges compared to industrial sensors, the validated bandwidth of the IMUA makes it possible to describe kinematics and dynamics of running activity. Moreover, the IMU features major advantages such as autonomy and portability, both crucial for sport purposes. This conclusion is in agreement with that of Reenalda et al. [42] who validate the use of an IMU for kinematic measurements during the practice of a marathon. IMU present a strong potential for outdoor running measurement compared to industrial sensors dedicated to measurements limited to a restricted environment. However, the importance of the use of less damped shoes, practice on a hard or sloping ground or the practice of different sport activities cannot be emphasized too much as to the enlargement of the vibration ranges. Consequently, measuring vibration using an IMUA could result in other limitations in these different configurations, but this study could significantly be improved by adding new parameters or test results from other activities. As outlined earlier, the validation of this type of sensor enables studying in a simple way and in outdoor conditions, running characteristics such as stride pattern, shock and damping effect or stride frequencies.
